# Cholesterol as a key player in amyloid β-mediated toxicity in Alzheimer’s disease

**DOI:** 10.3389/fnmol.2022.937056

**Published:** 2022-08-25

**Authors:** Vladimir Rudajev, Jiri Novotny

**Affiliations:** Department of Physiology, Faculty of Science, Charles University, Prague, Czechia

**Keywords:** amyloid β, Alzheimer’s disease, membrane, cholesterol, lipids, GM1, lipid rafts

## Abstract

Alzheimer’s disease (AD) is a neurodegenerative disorder that is one of the most devastating and widespread diseases worldwide, mainly affecting the aging population. One of the key factors contributing to AD-related neurotoxicity is the production and aggregation of amyloid β (Aβ). Many studies have shown the ability of Aβ to bind to the cell membrane and disrupt its structure, leading to cell death. Because amyloid damage affects different parts of the brain differently, it seems likely that not only Aβ but also the nature of the membrane interface with which the amyloid interacts, helps determine the final neurotoxic effect. Because cholesterol is the dominant component of the plasma membrane, it plays an important role in Aβ-induced toxicity. Elevated cholesterol levels and their regulation by statins have been shown to be important factors influencing the progression of neurodegeneration. However, data from many studies have shown that cholesterol has both neuroprotective and aggravating effects in relation to the development of AD. In this review, we attempt to summarize recent findings on the role of cholesterol in Aβ toxicity mediated by membrane binding in the pathogenesis of AD and to consider it in the broader context of the lipid composition of cell membranes.

## Introduction

The plasma membrane (PM) serves as the major communication interface where the cell receives and processes almost all signals from the environment. Homeostasis of membrane lipids, including cholesterol, is critical for normal brain function (Litvinov et al., 2018; Kao et al., 2020; McFarlane and Kedziora-Kornatowska, 2020). In the human body, cholesterol is mainly concentrated in brain tissue, where it forms nearly half of lipid molecules and the brain contains 23% of the body’s total cholesterol (15 mg/g tissue), although this organ accounts for only 2.1% of total body weight (Dietschy and Turley, 2004; Takamori et al., 2006; Das et al., 2014; Egawa et al., 2016). About 70% of the cholesterol in the brain is associated with myelin, 20% is present in glia, and 10% is present in neurons. Cholesterol is essential for neuronal function. A deficiency of cholesterol in neurons can lead to impaired signal transduction and synaptic degradation (Frank et al., 2008; Egawa et al., 2016; Loera-Valencia et al., 2019).

Alzheimer’s disease (AD) is strongly associated with the accumulation of neurofibrillary tangles of hyperphosphorylated tau (p-tau) and the aggregation of amyloid β-42 (Aβ42) in the brain, which can be observed many years before the disease manifestation (Braak and Braak, 1997; Braak et al., 2011; Tarawneh and Holtzman, 2012; Villemagne et al., 2013; Cho et al., 2016). Both tau and amyloid pathology are associated with neuroinflammation and neurodegeneration (Braak and Braak, 1997; Jack et al., 2010; Tonnies and Trushina, 2017; Vogels et al., 2020). Moreover, lipid dyshomeostasis is an obvious feature of all stages of AD (Panza et al., 2006; Chan et al., 2012; Naudi et al., 2015; de Oliveira et al., 2017; Agarwal and Khan, 2020). Since lipids are the basic constituents of cell membranes, changes in phospholipids, sphingolipids, cholesterol, and the degree of unsaturation of fatty acids have a nonnegligible impact on the action of amyloid peptides, blood-brain barrier (BBB) disruption, mitochondrial dysfunction, oxidative stress, inflammation, and neurodegeneration (Panza et al., 2006; Di Paolo and Kim, 2011; Agrawal et al., 2020; Chew et al., 2020; Kao et al., 2020).

Several genes implicated in the pathophysiology of AD are involved in cholesterol metabolism, including apolipoprotein E (ApoE), apolipoprotein J (clusterin), ATP-binding cassette (ABC) transporters A and G, and SORL1 (receptor for lipoprotein particles). Others, such as phosphatidylinositol-binding clathrin assembly protein (PICALM), CD2-associated protein (CD2AP), and bridging Integrator-1 (BIN1) are associated with membrane trafficking, which is also more or less cholesterol-dependent (Reitz, 2013; Kim et al., 2017; Pimenova et al., 2018; Kunkle et al., 2019; Abe-Dohmae and Yokoyama, 2021; Dai et al., 2021). ApoE, which is responsible for the majority of intercellular cholesterol transport in the brain, has the greatest relevance to AD pathology. ApoE exists in three variants, ApoE2, E3, and E4, with ApoE4 being the most risky allele in the sporadic form of AD (Hayashi et al., 2002; Morrow et al., 2002; Leoni et al., 2010; Youmans et al., 2012; Tai et al., 2013; Oikawa et al., 2014; Wood et al., 2014; Chang et al., 2017; Lin et al., 2018; Fernandez et al., 2019; Lanfranco et al., 2020; Lee et al., 2021; de Leeuw et al., 2022). Cholesterol does not pass through the BBB and nearly all brain cholesterol is synthesized in the brain, particularly in astrocytes, as the neuronal synthetic pathway is insufficient to meet the brain’s demand for this lipid. Therefore, ApoE-mediated cholesterol transport is of critical importance (Jurevics and Morell, 1995; Dietschy and Turley, 2004; Takamori et al., 2006; Czuba et al., 2017; Ferris et al., 2017).

On the other hand, when considering only cholesterol itself, a meta-analysis of genome-wide significant single nucleotide polymorphisms in AD patients confirmed no association between cholesterol and AD (McFarlane and Kedziora-Kornatowska, 2020; Nilsson et al., 2021). However, other systematic reviews found a broad association between many proteins involved in cholesterol transport and metabolism and AD (Agarwal and Khan, 2020; Xin et al., 2021). In this review, we would like to highlight the significance and also the controversy of the role of cholesterol in AD pathology related to amyloid β toxicity. Because cholesterol affects several processes associated with AD pathology including Aβ formation, transport, and degradation, we would like to focus specifically on the effects mediated by Aβ binding to cell membranes in relation to cholesterol content.

## The Interplay Between Cholesterol and AD

### Amyloid β

Aβ peptides are produced from the transmembrane amyloid precursor protein (APP). APP can be cleaved by α-secretase in the PM *via* the non-amyloidogenic pathway. On the other hand, mainly intracellular amyloidogenic processing by β- and γ-secretase leads to Aβ40/42 products (Xiong et al., 2008; Chow et al., 2010; Arbor et al., 2016). While Aβ40, a peptide of 40 amino acids, accounts for the majority of amyloid β in the brain, Aβ42, which is two hydrophobic amino acids longer, is considered the major neurotoxic amyloid peptide (Yip et al., 2002; Williams et al., 2010; Bode et al., 2017).

The metastability of many cellular proteins allows them to transform into more thermodynamically stable aggregates under appropriate conditions (Honeycutt and Thirumalai, 1990; Baldwin et al., 2011). The membrane environment may be a key factor in the formation of aberrant amyloid conformations of proteins rich in β-sheets, including Aβ42 (Straub and Thirumalai, 2014). Amyloids can organize into small annular oligomers, curvilinear protofibrils, and larger aggregates that form diffuse or dense plaques composed of fibrils of varying composition. Depending on the environment, they show different toxicity under different conditions (Bucciantini et al., 2014). Oligomeric Aβ is considered the most toxic form of amyloid, causing membrane perturbation, calcium dyshomeostasis, reactive oxygen species (ROS) production, mitochondrial and endosomal-lysosomal dysfunction (Williams et al., 2010; Narayan et al., 2014; Evangelisti et al., 2016; Oku et al., 2017; Tonnies and Trushina, 2017; Fernandez-Perez et al., 2018; Ciudad et al., 2020). Biological surfaces can catalyze the aggregation and growth of amyloid structures in ways that differ from nucleation in solution. In particular, cell membranes composed of miscellaneous lipids, including neutral and anionic lipids, polyunsaturated, monounsaturated, and saturated fatty acids, and cholesterol, provide a highly variable platform that offers a specific polar environment as well as a hydrophobic zone in which amyloid peptides can interact and aggregate into different forms with varying degrees of toxicity (Ding et al., 2012; Cecchi and Stefani, 2013). The way amyloid acts on cells always represent a complex interplay between Aβ and the specific membrane composition with which the amyloid interacts. Therefore, cytotoxicity must be considered as a novel feature resulting from the interaction between amyloid peptide and membrane (Bucciantini et al., 2014; Liu P. et al., 2015; Evangelisti et al., 2016).

### Cholesterol homeostasis affects brain health

In the brain, cholesterol is necessary for synaptogenesis, neuronal differentiation and plasticity, regeneration and myelinization, and normal neuronal function and morphology (Hussain et al., 2019). By inducing and stabilizing membrane curvature, cholesterol facilitates fusion of synaptic vesicles with the PM and is required for endocytosis, making neurotransmitter release and recycling highly dependent on cholesterol (Subtil et al., 1999; Egawa et al., 2016; Petrov et al., 2016). Cholesterol confers stability to lipid rafts and provides the basis for raft association of membrane receptors, channels, and other proteins in both healthy cells and in pathophysiological processes (Simons and Toomre, 2000; Anderson and Jacobson, 2002; Hicks et al., 2012; Egawa et al., 2016; Vona et al., 2021). Modifications of brain sterols, including cholesterol and oxysterols, can lead to disruption of ion homeostasis, alterations in neurotransmitter release, and propagation of excitotoxicity (Ong et al., 2010; Djelti et al., 2015). And, even if brain cholesterol levels do not change, decreased oxidation and efflux from the brain may follow decreased cholesterol biosynthesis, as observed in a transcriptome study of the hippocampus and entorhinal cortex of AD-affected individuals, but not in the visual cortex, which is less affected by neurodegeneration (Varma et al., 2021). Both tau and amyloid pathology are associated with cholesterol homeostasis and regulation, at least in part independently (van der Kant et al., 2020). Many data confirm the disruption of cholesterol metabolism in AD, as the major oxidation product of cholesterol, 24S-hydroxycholesterol, is elevated in the cerebrospinal fluid of AD patients (Leoni et al., 2006; Panza et al., 2006). On the other hand, the reduction of 24S-hydroxycholestrol, especially later in the course of AD, was observed (Heverin et al., 2004; Testa et al., 2016; Kao et al., 2020; Varma et al., 2021).

### Alterations in cholesterol content in the brain

Cholesterol levels increase by 19%–34% in the gray matter of the cerebral cortex in AD brains (Xiong et al., 2008; Lazar et al., 2013). However, other studies have reported a decrease in brain cholesterol content (Ledesma et al., 2003; Egawa et al., 2016). The controversial data might be related to the different cholesterol metabolism in different brain regions during aging and during neuropathological processes. Also, the different results linking cholesterol levels to AD often reflect the analysis of different tissues (blood, brain), brain parts (cerebral cortex, cerebellum, whole brain), cell populations (neurons, astrocytes), or membrane compartments (plasma membrane, lipid rafts; Martin et al., 2010; Ledesma et al., 2012). Moreover, it is not only the decrease or increase in the lipid that matters but also the magnitude of the change. While a slight decrease in cholesterol levels can be protective, a loss of more than 30% can lead to cell death (Martin et al., 2010). Although changes in cholesterol content, metabolism, and transport have been linked to AD (Kirsch et al., 2002; Runz et al., 2002; Burns et al., 2006; Pincon et al., 2015; Loera-Valencia et al., 2019), it is still controversial whether cholesterol plays a role in the onset of neurodegeneration or whether its dyshomeostasis is simply a consequence of preceding Aβ-promoted pathology. Moreover, the significance of the change is questionable, as cholesterol loss may represent a physiological process that helps neurons combat stress (Martin et al., 2011).

### Membrane structure and cholesterol-dependent lipid rafts

Lipid rafts are small (10–200 nm), heterogeneous and highly dynamic assemblies enriched in cholesterol and sphingolipids. The high content of saturated hydrocarbon chains allows raft lipids to form a liquid-ordered (Lo) phase characterized by lower fluidity and tight intermolecular contacts within the bilayer (Brown, 1998; Anderson and Jacobson, 2002; Lingwood and Simons, 2010; Rushworth and Hooper, 2010). Lipid rafts play a role in the processes of Aβ formation, aggregation, and interactions with membranes that lead to amyloid toxicity (Rushworth and Hooper, 2010; Hicks et al., 2012; Arbor et al., 2016). Because cholesterol is one of the key structural raft elements, its membrane concentration may have a major impact on Aβ-related pathological processes. Mature neurons have been shown to contain more lipid rafts, making them more susceptible to amyloid-induced damage and degeneration (Malchiodi-Albedi et al., 2010). In contrast to glial cells, neurons are characterized by high levels of complex gangliosides and sphingomyelin (SM), which support the formation and maintenance of rafts. Therefore, differences between different cells and brain parts may be an important factor in the propagation of Aβ-induced cytotoxicity (Abramov et al., 2011; Grassi et al., 2020).

Lipid rafts from the frontal and entorhinal cortex, but not from the cerebellum, of AD patients are more liquid-ordered than those of control subjects. This is not due to changes in cholesterol or SM levels (which were reduced in these rafts) but to a decrease in the unsaturation of fatty acids. The higher viscosity of lipid rafts is associated with increased β-secretase/APP interaction and higher Aβ production (Martín et al., 2010; Fabelo et al., 2014; Diaz et al., 2015, 2018). It is interesting to note that neurodegenerative diseases such as AD, dementia with Loewy bodies, or Parkinson’s disease share some changes in the composition of raft lipids but the individual diseases differ considerably in terms of specific changes in some lipids or fatty acids (Marin et al., 2017). Molander-Melin et al. (2005) observed a decrease in the content of lipid rafts in the temporal cortex of AD brains, indicating a change in overall membrane composition and physical properties. Moreover, lipid rafts from the frontal and temporal cortex were poor in cholesterol but enriched in gangliosides GM1 and GM2 (Molander-Melin et al., 2005). Aβ was also observed to affect tau phosphorylation in lipid rafts, with a link between Aβ and raft-associated tau population *via* the cdk5 phosphorylation pathway (Hernandez et al., 2009).

### Cholesterol distribution in cellular membranes

Cholesterol is concentrated in the PM of animal cells, where it is mainly localized in lipid rafts rich in SM (Das et al., 2014; Litvinov et al., 2018). In the endoplasmic reticulum (ER), cholesterol content reaches only about 1% and increases markedly during the secretory pathway (Maxfield and Wustner, 2002). The different cholesterol content in cell membranes is reflected in the different amounts of lipid rafts in which cholesterol is concentrated. The concentrations of “free” non-raft cholesterol are approximately the same in the ER and PM (Litvinov et al., 2018), but the amount of cholesterol in the ER exhibits greater variation than in the PM (Petrov et al., 2016).

In young mice, asymmetric cholesterol distribution within the bilayer has been observed, with most cholesterol associated with the inner leaflet of the synaptosomal plasma membrane (SPM), which is more rigid than the exofacial leaflet (Igbavboa et al., 1996). Changes in the distribution can occur without changes in the total amount of cholesterol in the brain. Interestingly, there is a shift of cholesterol from the cytofacial to the exofacial leaflet during aging or ethanol treatment (Wood et al., 1984, 1990, 2002; Viani et al., 1991; Igbavboa et al., 1996; Eckert et al., 2001; Kirsch et al., 2002). These results are in contrast to those of Molander-Melin et al. (2005), who observed decreased cholesterol and raft content during AD. However, aging need not correspond exactly to AD development, and synaptosomal membranes are not readily comparable to rafts isolated from the cerebral cortex in the Molander-Melin study.

Increased cholesterol in the outer layer of the membrane may be related to altered interactions of the cell with Aβ and other ligands that require cholesterol or less fluid membrane of lipid rafts. This has implications for signaling, ion transport, or endocytosis/exocytosis, all of which are impaired at AD (Wood et al., 2002, 2014). Because ApoE4 is less effective than ApoE2/3 in cholesterol export from the exofacial leaflet of the neuronal plasma membrane, the ApoE4 phenotype may lead to an increase in the cholesterol pool associated with the outer membrane layer. Then, the altered cholesterol content in the PM may affect Aβ binding and its toxic effects (Hayashi et al., 2002). Moreover, the presence of ApoE2 was found to be associated with lower cholesterol content in synaptic membrane microdomains, which may be related to lower amyloidogenic APP processing and lower levels of cholesterol- and GM1-dependent Aβ aggregation (Oikawa et al., 2014). It was concluded that the neuronal membranes of older individuals or carriers of the ApoE4 allele may be more susceptible to Aβ-mediated perturbations than those of younger individuals or carriers of the ApoE2 or ApoE3 alleles (Wood et al., 2014).

### Hypercholesterolemia as a risk factor for AD

Brain cholesterol levels are not directly influenced by higher plasma cholesterol levels because cholesterol cannot pass through the BBB. However, diet-induced hypercholesterolemia is associated with increased Aβ production and AD pathology (Ghribi et al., 2006; Oksman et al., 2006; Jaya Prasanthi et al., 2008; Wieckowska-Gacek et al., 2021). Thus, indirect mechanisms following atherosclerosis or cerebrovascular damage may play a role in cholesterol-related AD pathology (Hooijmans et al., 2007; Anstey et al., 2017). High plasma cholesterol, especially in midlife, perturbs cholesterol homeostasis and is considered a risk factor for AD (Kivipelto et al., 2001; Yaffe et al., 2002; Solomon et al., 2009; Ricciarelli et al., 2012; Tarawneh and Holtzman, 2012; Schilling et al., 2017; Wang et al., 2020).

In a transgenic mouse model of AD (TgAPPsw), a high-cholesterol diet for 7–10 months resulted in increased deposition of Aβ plaques in the brain (Shie et al., 2002). Hypercholesterolemia induced by a high-cholesterol diet increased Aβ content in the temporal cortex of rabbits, where ApoE levels were also increased, indicating a link between plasma and brain cholesterol pools (Wu et al., 2003). Administration of a high-cholesterol diet to rats resulted in decreased cognitive performance, increased neuroinflammatory markers, p-tau, altered hippocampal morphology, including atrophy, and increased microglial cell activation (Ledreux et al., 2016; Jin et al., 2018). Similar results were obtained in the hypercholesterolemic AD model of transgenic mice (Umeda et al., 2012). The high-fat diet with cholesterol did not increase brain cholesterol levels, but the increase of Aβ deposition in plaques was observed in the brains of APP23 model mice (Fitz et al., 2010). Thus, disruption of cholesterol homeostasis can lead to serious health problems and a higher risk of AD, especially if cholesterol levels fluctuate over the years (Chung et al., 2019).

Neurofibrillar tangle-bearing neurons have been shown to contain more intracellular free cholesterol than adjacent tangle-free neurons in human AD brains (Distl et al., 2001). Extracellular colocalization of cholesterol, fibrillar amyloid plaques, and ApoE was demonstrated by Burns et al. (2003). In a transmission electron microscopy study, the interaction of cholesterol with the 17–21 hydrophobic cholesterol-binding motif of Aβ fibrils was observed (Harris, 2008). In hypercholesterolemic AD model APP/PS1/SREBP-2 transgenic mice, the amount and toxic effects of Aβ42 including tau pathology, oxidative damage, and neuroinflammation, were increased compared with APP/PS1 mice with normal cholesterol levels (Barbero-Camps et al., 2013). Increasing plasma membrane cholesterol levels by 30% in cultured neurons led to AD pathology, including enlargement of early endosomes and increased Aβ42 production (Marquer et al., 2014). On the other hand, lowering cholesterol content by hydroxypropyl-β-cyclodextrin had a neuroprotective effect in Tg19959 mice (Yao et al., 2012). The experiments on cultured hippocampal neurons indicated that mature neurons were more susceptible to Aβ-induced calpain cleavage of tau, disruption of calcium homeostasis, and cell death than the young cells. Neuron maturation was accompanied by a developmental increase in membrane cholesterol levels. When cholesterol levels were lowered in mature neurons, the toxic effect of amyloid was reduced. Conversely, an increase in membrane cholesterol in young cells enhanced their susceptibility to Aβ insult (Nicholson and Ferreira, 2009).

Impairment of mitochondria and altered mitophagy are among the features of AD (Kerr et al., 2017; Fang et al., 2019; Tran and Reddy, 2021). Intraneuronal cholesterol accumulation can disrupt autophagy, reduce mitophagy, induce mitochondrial oxidative stress, and inhibit lysosomal degradation of Aβ, leading to AD progression (Roca-Agujetas et al., 2021). Mitochondrial cholesterol accumulation also impairs antioxidant glutathione (GSH) import into mitochondria, leading to increased mitochondrial oxidative stress and enhanced Aβ neurotoxicity in the APP/PS1 transgenic mouse model of AD (Fernandez et al., 2009). High brain cholesterol impaired the fusion of autophagosomes with the endosomal-lysosomal compartment, leading to impaired tau and Aβ degradation (Barbero-Camps et al., 2018). *In vitro* studies showed that elevated cellular cholesterol disrupted endosomal and lysosomal Aβ degradation and resulted in amyloid accumulation in Neuro2a cells (Takeuchi et al., 2019).

Statins lower cholesterol levels by inhibiting the key regulatory enzyme in cholesterol synthesis, 3-hydroxy-3-methylglutaryl-CoA reductase (Fassbender et al., 2001; Kirsch et al., 2003). Although some results do not confirm the beneficial effects of statins in terms of AD development (Ott et al., 2015; de Oliveira et al., 2017; Schultz et al., 2018; Williams et al., 2020), numerous data suggest improvement in cognitive and memory functions and reduction in the risk of developing dementia (Jick et al., 2000; Wolozin et al., 2000; Yaffe et al., 2002; Green et al., 2006; Carlsson et al., 2008; Kurinami et al., 2008; Haag et al., 2009; Lin et al., 2015; Xuan et al., 2020). However, statins exhibit many pleiotropic effects that are not always related to lowering cholesterol (Pedrini et al., 2005; Ostrowski et al., 2007; Won et al., 2008; Cheng S. W. et al., 2013; Daneschvar et al., 2015; Sun et al., 2015; Jeong et al., 2021).

### Altered cholesterol levels may modulate Aβ action

Some studies do not support the assumption that AD is associated with hypercholesterolemia. The results show that brain cholesterol levels vary widely among AD patients, and they do not support the idea that total brain cholesterol abundance is a causative factor in AD (Mielke et al., 2005; Panza et al., 2006; Wood et al., 2014). A recent study by Bennett et al. (2020) found no association between total cholesterol, LDL cholesterol, HDL cholesterol, and triglycerides in midlife and amyloid pathology later in life. However, low serum cholesterol concentration has been associated with impaired cognitive performance and preceded the onset of AD (Elias et al., 2005; Stewart et al., 2007).

Aging has been found to lead to decreased cholesterol synthesis in astrocytes, which may contribute to the synaptic and neuronal degeneration associated with senescence (Boisvert et al., 2018). In addition, oligomeric Aβ inhibits cholesterol synthesis, as observed in mouse cerebrocortical cells (Mohamed et al., 2018). GT1–7 hypothalamic cells were manipulated to decrease cholesterol production, which resulted in increased apoptosis in the presence of Aβ (Fukui et al., 2015). In membranes isolated from AD brains, a negative correlation was observed between cholesterol content and susceptibility to the disruptive effects of Aβ (Eckert et al., 2000). This suggests a protective effect of cholesterol in Aβ exposed cells, which was confirmed by the study performed on mouse neuronal membranes (Kirsch et al., 2002).

### Cholesterol-dependent Aβ production

Lipid rafts provide an optimal environment for Aβ production (Wahrle et al., 2002; Silva et al., 2013). Amyloidogenic cleavage of APP is mediated by β- and γ-secretase and results in the deleterious Aβ40–42 products prone to aggregation (Fraering et al., 2004; Xiong et al., 2008; Chow et al., 2010; Arbor et al., 2016). Both β- and γ-secretases show higher activity in cholesterol-rich lipid rafts. Hence, higher cholesterol content ensures optimal conditions for their activity and likely leads to stabilization of β- or γ-secretases, enhancing their enzymatic activity and reducing their degradation (Wahrle et al., 2002; Cordy et al., 2003; Fraering et al., 2004; Kalvodova et al., 2005; Osenkowski et al., 2008; Xiong et al., 2008; Beel et al., 2010; Sathya et al., 2017). Under non-pathological conditions, non-amyloidogenic α-secretase cleavage predominates and is localized in the more fluid non-raft cholesterol-poor membrane (Kojro et al., 2001; Beel et al., 2010; Chew et al., 2020). It has been shown that α-secretase activity is inhibited by cholesterol (Bodovitz and Klein, 1996).

In contrast to secretases, APP shows a more dynamic cellular localization. APP has been found to be localized in the PM, endocytic compartment, ER, and Golgi apparatus (GA). Moreover, specific APP distribution in sub-compartments such as mitochondria-associated ER, trans-GA, or lipid rafts is strongly associated with differential APP processing (Lee et al., 1998; Kawarabayashi et al., 2004; Fabiani and Antollini, 2019). For amyloidogenic cleavage, the APP must be colocalized in a cholesterol-dependent manner with β- and γ-secretases in the intracellular lipid raft membrane (Ehehalt et al., 2003; von Arnim et al., 2008; Area-Gomez et al., 2009; Cossec et al., 2010; Panahi et al., 2016; Chung et al., 2018; DelBove et al., 2019). Since cholesterol interacts directly with APP and secretase actions also depend on cholesterol, the cleavage of APP and the resulting amyloid production are strongly linked to the amount of cholesterol and its membrane distribution (Marquer et al., 2011; Barrett et al., 2012; Decock et al., 2015; Kim et al., 2016; Sun et al., 2017; Audagnotto et al., 2018; Agrawal et al., 2020; Montesinos et al., 2020; Pantelopulos et al., 2020; Nierzwicki et al., 2021).

Thus, cholesterol content controls the amyloidogenicity of APP processing, but on the other hand, Aβ reduces both cholesterol production and uptake from the surrounding environment through a negative feedback loop (Beel et al., 2010). Moreover, APP may serve as a cholesterol-sensitive and regulatory element (Pierrot et al., 2013). In addition to the production machinery, enzymes involved in Aβ degradation (insulin-degrading enzyme, neprilysin, endothelin-converting enzymes, plasmin) have also been shown to localize to cholesterol-rich lipid rafts. Hence, cholesterol plays an important role not only in amyloidogenic APP processing but also in more complicated regulatory pathways (Sun et al., 2015). In the following sections, we would like to focus specifically on Aβ-mediated effects related to the cholesterol pool localized in the plasma membrane of brain cells.

## Amyloid β Toxicity Related to Membrane Cholesterol

### Membranes as nucleation platforms for Aβ aggregation

**Figure 1:**
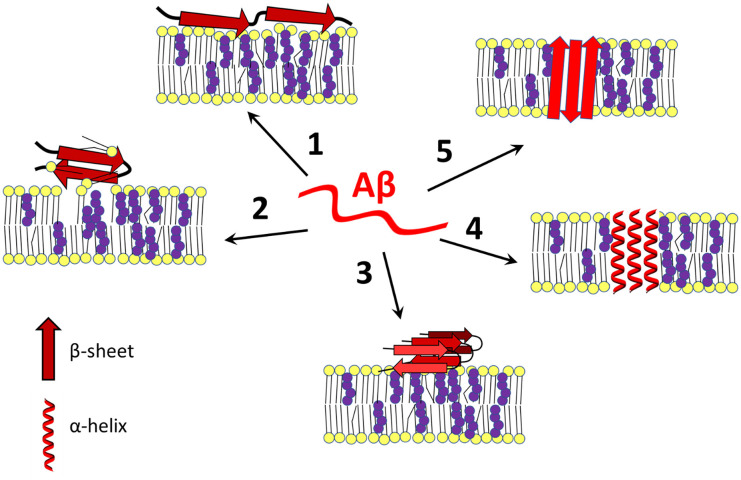
Amyloid β-membrane interactions. Amyloid β interacts with the plasma membrane in different ways, leading to disruption of cell homeostasis. Aβ exists in a disordered state (in the center) or can change its conformation to an α-helical or β-sheet containing form after contact with membrane lipids (especially cholesterol-rich lipid rafts), Aβ binds to the surface of the bilayer and destabilizes the membrane by a carpeting effect (1), or it may act as a detergent (2). In addition, the membrane may serve as a platform for aggregation of Aβ in its most harmful β-sheet-rich conformation (3). Under certain conditions, with or without the help of cholesterol, Aβ self-organizes into an α-helical (4) or β-barrel (5) transmembrane pore that is often selective for calcium ions. Changing cholesterol content and leaflet distribution may cause Aβ to transition from the surface-bound to the transmembrane form and *vice-versa* (not shown). Red—amyloid β, yellow—membrane lipids, purple—cholesterol. The scheme was prepared according to Eckert et al. (2001); Yip et al. (2002); D’Errico et al. (2008); Abramov et al. (2011); Zhao et al. (2011); Drolle et al. (2012); Yu and Zheng (2012); Fantini et al. (2014); Seghezza et al. (2014); Brown and Bevan (2016); Press-Sandler and Miller (2018); Staneva et al. (2018); Fabiani and Antollini (2019); Qu et al. (2019); and Banerjee et al. (2021).

As mentioned above, amyloid peptides adopt different spatial arrangements depending on their surroundings. After interaction with a membrane, Aβ can damage the bilayer through a “carpeting effect” in which the amyloid disrupts membrane integrity by covering its surface, it can exert detergent-like effects, or amyloid peptides can self-organize into transmembrane ion channels (Figure 1; Williams and Serpell, 2011; Cecchi and Stefani, 2013; Press-Sandler and Miller, 2018; Sciacca et al., 2018). Due to the presence of different nucleation centers, the nature of the lipid membrane determines the state of the peptide, which is closely linked to the processes of Aβ assembly and toxicity (Ding et al., 2012; Cecchi and Stefani, 2013; Pannuzzo, 2016; Bera et al., 2019; Qu et al., 2019).

Even pathophysiological amyloid concentrations in the brain are too low to readily initiate self-aggregation (Hu et al., 2009; Arbor et al., 2016). Therefore, hetero nucleation using non-amyloid organizing centers must be at the beginning of the aggregation process. The polar and charged N-terminal region of Aβ binds to polar heads of membrane lipids, whereas the hydrophobic C-terminal region is responsible for nonpolar interactions with the hydrophobic zone containing cholesterol. Although both parts are distinct, they can interact cooperatively, especially in a hydrophilic/hydrophobic membrane environment (Srivastava et al., 2019). Since the molecular arrangement of Aβ strongly depends on the oligomerization process, the particular structure of the nucleation centers, their membrane density and distribution, and disease- or age-dependent alterations play a crucial role in AD (Matsuzaki, 2014; Amaro et al., 2016; Matsubara et al., 2017; Azouz et al., 2019; Srivastava et al., 2019).

In the Tg2576 transgenic mouse model of AD and in the brains of AD patients, Aβ was highly concentrated in lipid rafts along with ApoE and p-tau, suggesting that lipid rafts serve as sites where external Aβ communicates with internal elements involved in the pathology of AD (Kawarabayashi et al., 2004). Brain membranes from naked mole rats rich in cholesterol and lipid rafts were found to be more susceptible to Aβ-mediated perturbations, but these long-lived animals showed increased resistance to oxidative stress, which could also be attributed to their highly organized and polyunsaturated fatty acid (PUFA)-poor membranes (Frankel et al., 2020). When Aβ42 is bound to rafts, it can be endocytosed but does not reach lysosomes and accumulates intracellularly in neurons. In contrast, lowering cholesterol levels with squalestatin was connected with a decreased association of Aβ42 with rafts and increased lysosomal degradation of amyloid peptide (Simmons et al., 2014). An atomic force microscopy (AFM) study of the Lo/Ld (liquid-disordered) membrane model observed specific targeting of Aβ to the disordered phase, but specifically to the boundaries between Ld and Lo. These boundaries represent sites of higher hydrophobic mismatch and lower stability that may help Aβ mediate its neurotoxic defects (Azouz et al., 2019).

### Amyloid β-cholesterol interactions

The interaction of Aβ with cholesterol in the PM and lipid rafts plays a role in Aβ seeding, aggregation, and toxicity (Mizuno et al., 1999; Yanagisawa, 2005; Schneider et al., 2006; Qiu et al., 2009). The binding of Aβ to cholesterol has been observed in senile plaques in the brain, where cholesterol accumulated along with ApoE (Panchal et al., 2010). Molecular dynamics (MD) simulations led to the conclusion that cholesterol facilitates Aβ membrane binding by making the interaction more energetically favorable. Cholesterol increases surface hydrophobicity, promotes more ordered lipid packing, and reduces lipid mobility. Then, Aβ binds preferentially to the cholesterol-rich regions of the artificial lipid bilayers (Yu and Zheng, 2012).

Studies on model membranes have shown that Aβ interacts only with lipid layers containing cholesterol (Avdulov et al., 1997; Henry et al., 2018). Small, but not large aggregates of Aβ(25–35) interacted with a model lipid monolayer composed of SM, 1-palmitoyl-2-oleoylphosphatidylcholine (POPC), and cholesterol (Cuco et al., 2016). The interaction was enhanced under acidic conditions, which corresponds to endosomal environment in which Aβ cytotoxic effects are expected to occur after Aβ endocytosis (Hu et al., 2009; Cuco et al., 2016). Depletion of cholesterol from the endosomal/lysosomal compartment reduced Aβ toxicity and Aβ aggregation in lysosomes of wild-type mice and AD model TgCRND8 mice (Yang et al., 2017). Lowering cholesterol level by 30% reduced Aβ cytotoxicity and increasing cholesterol content by 30% increased Aβ cytotoxicity in neuron-like PC12 cells. Association of amyloid with cholesterol and GM1-rich raft membranes was associated with Aβ aggregation, shift to a β-sheet-containing form, and toxicity (Lin et al., 2008; Matsuzaki, 2011; Mori et al., 2012).

It appears that cholesterol can promote or inhibit Aβ aggregation at the membrane, affect the secondary structure of amyloid and its ability to penetrate the bilayer (Williams and Serpell, 2011; Yu and Zheng, 2012). Many factors influence the outcome of the interaction: the molar ratio of lipid/cholesterol in the cell membrane, the presence of anionic lipids, SM, and gangliosides (especially GM1), lipid membrane ordering and fluidity, the amyloid species, the extent of Aβ oligomerization, the pH, the presence of other proteins or amyloid membrane receptors (Yu and Zheng, 2012; Meleleo et al., 2013; Dies et al., 2014; Amaro et al., 2016; West et al., 2017; Owen et al., 2018; Carrotta et al., 2021; Smeralda et al., 2021; Wiatrak et al., 2021). Moreover, physical parameters such as macromolecular crowding or vesicle size and membrane curvature play a role in Aβ–membrane interaction (Hirai et al., 2018; Terakawa et al., 2018). Also, phosphorylation of Aβ42 affects amyloid aggregation, interaction with the cholesterol-containing lipid bilayer, and toxicity of the peptide (Jamasbi et al., 2017). It must be emphasized that Aβ40 and Aβ42 differ significantly in their interaction with membranes. Aβ42 exhibits a higher ability to bind the bilayer or form Ca^2+^-selective transmembrane pores and shows more complex behavior than Aβ40 (Yip et al., 2001; Williams et al., 2010; Phan et al., 2013; Bode et al., 2017; Carrotta et al., 2021).

### Cholesterol supports the formation of transmembrane Aβ pores

Amyloid β peptides have been shown to oligomerize into ion-permeable transmembrane pores of varying structure, composition, and permeability. Aβ42 oligomers are known to adopt a β-sheet-rich structure in the presence of membrane lipids and self-organize into transmembrane channels permeable to calcium ions, which can cause cell death (Prangkio et al., 2012; Arbor et al., 2016; Serra-Batiste et al., 2016; Lee et al., 2017; Julien et al., 2018; Ciudad et al., 2020; Ruiz-Arias et al., 2020; Venko et al., 2021).

Mass spectrometry and circular dichroism measurements showed that cholesterol supported the incorporation of Aβ into lipid vesicles. In contrast to the above results, Aβ incorporation resulted in increased α-helicity of the Aβ peptide. However, under cholesterol-depleted conditions, most of Aβ40 on the vesicle surface remained in a β-sheet-rich, aggregation-prone conformation (Ji et al., 2002). Cholesterol also induced α-helical Aβ topology in transmembrane annular octameric channels, as shown in MD simulations and SH-SY5Y cell culture studies (Di Scala et al., 2014, 2016). In Aβ, the linear sequence of amino acids 22–35 is a functional cholesterol-binding domain that is unusual in that it does not contain the aromatic residues common in other cholesterol-binding protein domains. The Aβ–cholesterol interaction may promote Aβ incorporation and the formation of α-helical amyloid pores in cholesterol-rich lipid rafts (Di Scala et al., 2013).

In another study, Aβ40 aggregated on a bilayer surface containing SM but could insert into the artificial membrane only in the presence of cholesterol (Devanathan et al., 2006). Also, Aβ–monolayer interaction experiments and an MD-based study showed that the oligomerization process of Aβ42 and Aβ25–35 peptides in calcium-permeable pores was cholesterol-dependent. Bexarotene blocked the formation of Aβ-channels by preventing the binding between cholesterol and amyloid peptides (Fantini et al., 2014). Astrocytes were found to contain a higher amount of cholesterol in their PM than neurons, which was associated with a higher extent of Aβ incorporation into membranes and increased calcium influx into cells (Abramov et al., 2011; Angelova and Abramov, 2017). Cholesterol affects Aβ conformation and aggregation through both direct interaction and modulation of membrane structure, as described in the study of calcium-permissive amyloid membrane pore formation (Kandel et al., 2019).

### Cholesterol impedes Aβ incorporation into the bilayer but promotes Aβ binding at the membrane surface

The presence of cholesterol has been demonstrated to inhibit Aβ association with the membrane and stabilize membranes (Yip et al., 2001; Phan et al., 2013). In the MD model, cholesterol blocks amyloid pore formation by binding to Aβ42 (Zhao et al., 2011). Cholesterol may have a protective effect on Aβ action, as higher cholesterol levels have been associated with increased membrane ordering and rigidity, which is connected with a reduced ability of amyloid to enter the bilayer and alter membrane properties (Eckert et al., 2001; Seghezza et al., 2014; Staneva et al., 2018; Fabiani and Antollini, 2019).

Besides affecting the incorporation into the bilayer, cholesterol may also determine the binding of amyloid to the membrane surface. Increased cholesterol resulted in increased binding of Aβ42 to the membrane surface in planar bilayers composed of different brain lipids, which was not associated with bilayer disruption (Yip et al., 2002). Cholesterol-containing lipid membranes promoted Aβ42 aggregation up to 20-fold through a surface-catalyzed nucleation process (Habchi et al., 2018).

MD simulations of Aβ42 tetramer binding to pure POPC or cholesterol-rich raft model membranes showed that cholesterol modulates Aβ fibril formation by reducing the extent of Aβ42 tetramer insertion into the bilayer and inducing intermolecular rearrangement of amyloid oligomers (Brown and Bevan, 2016). AFM visualization and MD simulations demonstrated that cholesterol in the lipid bilayer significantly increased Aβ42 surface aggregation, but not membrane penetration, at monomer concentrations as low as nM. These results suggest the importance of membrane lipids for the local concentration, clustering, and conformation change of Aβ into a β-structure-rich form of amyloid peptides (Drolle et al., 2012; Qu et al., 2019; Banerjee et al., 2021). It is speculated that the AD-associated elevation of cholesterol levels in the PM may increase the likelihood of membrane-dependent Aβ42 aggregation (Banerjee et al., 2021).

Aβ(25–35) was able to intercalate into the lipid bilayer only in the absence of cholesterol. When cholesterol was added, the monomers of the amyloid peptide were excluded from the bilayer (Dante et al., 2006). Electron paramagnetic resonance (EPR) spectroscopy analysis revealed a dual effect of cholesterol on the Aβ(25–35)-membrane interaction. Low cholesterol content favors penetration of the Aβ(25–35) peptide into the membrane, resulting in membrane stiffening and redistribution of cholesterol into the outer leaflet of the membrane. However, when cholesterol was enriched or redistributed into the outer leaflet, the rigid lipid raft-like membrane prevented the amyloid peptide from entering the bilayer (D’Errico et al., 2008). This result is consistent with the fact that raft composition, cholesterol amount, and distribution change during aging or neurodegeneration, as mentioned previously (Wood et al., 1984, 1990, 2002; Igbavboa et al., 1996; Eckert et al., 2001; Diaz et al., 2018).

### Protective role of cholesterol in Aβ-induced toxicity

**Figure 2:**
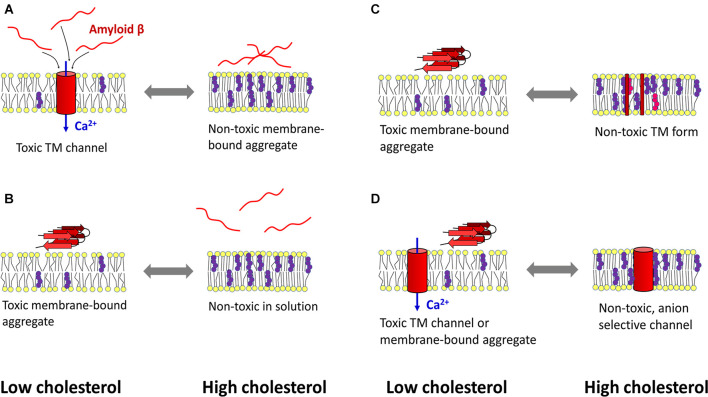
Mechanisms of cholesterol-mediated protection against amyloid β toxicity. **(A)** Left: under cholesterol-deficient conditions, amyloid β (Aβ) penetrates the membrane and aggregates in calcium-selective pores, leading to calcium dyshomeostasis and cytotoxicity. Right: cholesterol-induced rigidization of the membrane prevents amyloid peptide from entering the bilayer. **(B)** Another mechanism designates cholesterol as a lipid that reduces the ability of Aβ to bind the membrane. **(C)** A cholesterol-rich membrane (which may also contain oxysterols) retains Aβ peptides in a transmembrane (TM), non-toxic form. At low cholesterol levels, Aβ can self-organize into toxic aggregates at the membrane surface. **(D)** Aβ forms calcium-selective TM channels when the bilayer has low cholesterol content, whereas high cholesterol concentration induces the formation of non-toxic, anion-selective pores. Red—amyloid β, yellow—membrane lipids, purple—cholesterol, pink—oxysterol, blue—calcium influx into the cytosol. The scheme was prepared according to Arispe and Doh (2002); Curtain et al. (2003); Micelli et al. (2004); Qiu et al. (2009); Qiu et al. (2011); Meleleo et al. (2013); Evangelisti et al. (2014); Phan et al. (2014); Phan et al. (2018); Bode et al. (2017); Fernandez-Perez et al. (2018); and Meleleo et al. (2019).

As indicated above, cholesterol can protect cells from Aβ-induced membrane perturbations and cytotoxic effects (Figure 2). Increasing cholesterol levels in lysosomes led to decreased ROS production, decreased Aβ accumulation in the lysosomal compartment, and increased membrane stability (Oku et al., 2017). An atomistic MD simulation revealed a protective role of cholesterol in preventing membrane surface-induced amyloid-β sheet formation and Aβ42-induced bilayer disruption (Qiu et al., 2011). Enrichment of PC12 cells with cholesterol made the cells resistant to the calcium-mediated cytotoxic effect of Aβ, whereas reduction of membrane cholesterol enhanced the harmful effect of amyloid (Arispe and Doh, 2002). Similar results were obtained in experiments with fibroblasts isolated from familiar AD patients, rat brain cortical neurons, and SH-SY5Y neuroblastoma cells. There, increasing cholesterol levels decreased Aβ assembly into membrane-perturbating Ca^2+^-selective channels (Evangelisti et al., 2014). Using EPR and circular dichroism spectroscopy Curtain et al. found that membrane cholesterol reduced the ability of Aβ to enter the lipid bilayer and organize itself into α-helical transmembrane pores. However, Aβ that could not insert into the bilayer formed β-sheet structures on the membrane surface (Curtain et al., 2003). Based on results in cultured hippocampal neurons, Fernandez-Perez et al. (2018) proposed a model in which cholesterol plays a neuroprotective role. An increase in membrane cholesterol content prevents Aβ from incorporating into the bilayer and disrupting cellular homeostasis, while the peptide remains aggregated on the cell surface in large, nontoxic clusters. On the other hand, the amyloid peptide formed toxic membrane pores after cellular cholesterol levels were lowered by methyl-β-cyclodextrin (Fernandez-Perez et al., 2018).

Model membranes composed of POPC yielded a similar result: cholesterol decreased Aβ nucleation but promoted fibrilization on existing Aβ clusters. Moreover, a decrease in cholesterol level enhanced the association of Aβ42 with T-cell membranes. Increasing cholesterol levels in model membranes suppressed Aβ42-Ld interaction but enhanced Aβ42-Lo association, albeit to a lesser extent than Aβ42-Ld under lower cholesterol concentration conditions (Phan et al., 2014, 2018).

Another mechanism of the protective effect of membrane sterols may lie in the variability of ion selectivity of Aβ42-formed pores. The channels formed in the presence of oxidized cholesterol were anion-selective. Thus, oxysterols may serve as a protective mechanism against calcium-permeable pores formed by Aβ in model membranes and physiological membranes of cellular origin (Meleleo et al., 2013; Bode et al., 2017). The formation of Aβ40 channel assisted by oxysterols may also provide a protective mechanism against Aβfibrilization at the membrane surface (Micelli et al., 2004). Another mechanism has been proposed for metal ion-mediated amyloid toxicity. Hg^2+^ (and Pb^2+^) ions bind Aβ, which promotes β-structure formation and peptide aggregation. At the same time, the metals inhibit the interaction of Aβ with cholesterol, which otherwise traps the amyloid peptide in a nontoxic form in the membrane (Meleleo et al., 2019).

### Amyloid β affects membrane ordering and fluidity

The incorporation of amyloid aggregates resulted in membrane thinning and was accompanied by a 0.2 nm outward shift of the sterols and functional alterations in membrane lipid order (Ashley et al., 2006). In model systems, a strong association of Aβ42 with negative membrane lipids, including phospholipids, leads to membrane thinning related to Aβ aggregation and toxicity (Dong et al., 2017). Aβ(25–35) added to model membranes rich in anionic lipids displaced cholesterol molecules from the bilayer (Dies et al., 2014). A study performed on living cells showed that Aβ40 interacts with GM1 and decreases bilayer fluidity, whereupon β-secretase accelerates proteolytic cleavage of APP. This leads to a positive feedback loop in which Aβ stimulates its own production (Peters et al., 2009). In an MD-based study, polar Aβ residues, including Arg5, intercalated into the layer of polar lipid groups that stiffen the membrane. However, cholesterol and GM1 attenuated the extent of the perturbation and reduced the effect of Aβ42 on the model membrane (Brown and Bevan, 2017).

Cerebellar membranes were found to be more fluid and contain less cholesterol than cortex and hippocampus membranes, which were more susceptible to Aβ-induced destabilization. There, Aβ40 had a fluidization effect that was more pronounced in cholesterol-rich hippocampal and cerebral membranes (Chochina et al., 2001). Anionic artificial membranes containing 30%–40% cholesterol bound Aβ42, but this interaction was followed by increased cholesterol solubilization and decreased cholesterol plaque formation (Barrett et al., 2015). In model bilayers, Aβ bound to the rigid gel phase in the absence of relevant cholesterol content, whereas increasing cholesterol concentration to physiological levels resulted in decreased Aβ-membrane interaction and reduced membrane thinning and disturbances induced by amyloid (Choucair et al., 2007; Seghezza et al., 2014). Another study showed that after cholesterol depletion, the more fluid membranes were more sensitive to Aβ42-induced stabilization of lipid head interaction, resulting in membrane rigidization (Yip et al., 2002). Large unilamellar vesicles containing cholesterol and GM1 were stiffened by interaction with Aβ, which may have consequences for signal transduction and other processes that depend on the lipid raft environment (Hirai et al., 2013). In an MD simulation experiment, Aβ42 caused membrane perturbation through a carpeting effect connected with the formation of a more rigid, gel-like lipid phase; however, this effect was attenuated in the presence of cholesterol (Brown and Bevan, 2017).

Aβ bound to the non-raft Ld phase of model lipid bilayers, where Aβ, when GM1 was present, caused thickening and rigidization of the membrane. But when GM1 was absent in the Ld phase, a decrease in packing and thickness was observed (Staneva et al., 2018). These data show a strong dependence of amyloid-mediated effects on membrane composition, which must always be taken into account. Moreover, in SH-SY5Y neuroblastoma cells, Aβ42 induced thinning of rafts that altered their physicochemical properties and membrane perturbations only in more fluid, cholesterol-depleted membranes, indicating a protective effect of cholesterol against Aβ42 toxicity (Cecchi et al., 2009).

### Controversy about cholesterol-mediated protection against Aβ adverse effects

There are many data suggesting a protective role of cholesterol, but close attention must be paid to the precise arrangement of the experiments. In most studies, only Aβ monomers, truncated amyloid peptides, and artificial membranes lacking many important components, e.g., SM, gangliosides, or proteins, were analyzed (Avdulov et al., 1997; Dante et al., 2006; D’Errico et al., 2008; Qiu et al., 2011; Dies et al., 2014; Fantini et al., 2014; Brown and Bevan, 2016, 2017; Cuco et al., 2016). Moreover, the model membrane usually represents a nonphysiological phase arrangement, including gel-like anionic lipid bilayers (Dies et al., 2014). Since Aβ(25–35) contains only 11 amino acids, the preference for the thinner Ld membrane and the toxic effect exerted by the formation of β-sheet-rich ion channels only in this bilayer could be a consequence of the insufficient length of the peptide for spanning thicker membrane containing cholesterol (Lin and Kagan, 2002; Dante et al., 2006). In a study by Arispe and Doh (2002), Aβ toxicity was measured as a change in Ca^2+^ balance associated with the formation of transmembrane ion channels from the amyloid peptide. Nevertheless, when cholesterol was increased, Aβ was excluded from the membrane interior. Then, Aβ could remain on the surface of the bilayer in the toxic β-sheet-rich conformation, contradicting the protective role of cholesterol in Aβ-induced neurodegeneration when measured only as a level of calcium dyshomeostasis (Arispe and Doh, 2002; Curtain et al., 2003). In this context, it is important to emphasize that Aβ mediates its neurotoxicity in both transmembrane and surface-bound form. This suggests a high degree of complexity in the effects mediated by the cholesterol-Aβ interaction. This was demonstrated in a study by Liu R. Q. et al. (2015), in which cholesterol depletion in human SK-N-SH neuroblastoma cells led to decreased Aβ membrane incorporation and Ca^2+^-permeable channel formation, but also decreased Aβ degradation, increased Aβ aggregation and adsorption to the membrane, which was associated with higher amyloid toxicity (Liu R. Q. et al., 2015).

### Cholesterol distribution in the membrane bilayer affects Aβ behavior

A model shows that depletion of cholesterol from the exofacial leaflet and increased cholesterol content in the cytofacial leaflet thermodynamically favor membrane retention of a fully embedded Aβ peptide with an α-helical conformation. However, when cholesterol concentration decreases in the cytofacial leaflet and increases in the exofacial layer, which is typical of aging or AD and ApoE4-knock-in mouse synaptosomes (Wood et al., 1984, 1990, 2002; Igbavboa et al., 1996; Hayashi et al., 2002; Yanagisawa, 2005), the peptide loses the α-helicity and extrudes its reactive N-terminus into the extracellular space, which can lead to deleterious aggregation. Moreover, at very low membrane cholesterol, all Aβ is excluded from the bilayer and concentrates on the membrane surface (Liguori et al., 2013). MD simulations have shown that asymmetric cholesterol distribution in the Ld phase is associated with aggregation of Aβ monomers into membrane-spanning oligomers and bending of the bilayer, leading to vesiculation. In contrast, increased Lo phase rigidity causes Aβ to move toward the membrane-water interface (Pannuzzo, 2016).

AFM measurements revealed complex effects of cholesterol content on Aβ ion channel formation. Aβ42 is organized into ion channel structures in an artificial lipid bilayer with 15% cholesterol but not with 50% cholesterol or without cholesterol (Gao et al., 2020). This suggests a strong dependence of amyloid toxicity on cholesterol content, but the presence of other amyloid-binding partners, including membrane lipids and proteins must also be considered.

## The Involvement of Other Lipids

### Alterations in membrane lipids in AD

Cellular and especially neuronal membranes are complex structures, whose composition defines their function that is significantly affected by Aβ action. Although it is difficult to find the key factors responsible for amyloid-induced neuronal damage, it is certain that lipid ordering and membrane viscosity play important roles in both the amyloidogenic processing of APP and the toxic effects of Aβ (Cordy et al., 2003; Kalvodova et al., 2005; Osenkowski et al., 2008; Martín et al., 2010; Hicks et al., 2012; Fernandez-Perez et al., 2018; Srivastava et al., 2019). In this regard, fatty acids, sphingolipids, and cholesterol are the main players that, in addition to their general effect on membrane fluidity, show the ability to interact specifically with Aβ and contribute to the resulting action of this malignant peptide. In addition to cholesterol and sphingolipids, other lipids also play a role in AD pathogenesis, e.g., alterations in brain fatty acids including PUFAs, plasmalogens, sulfolipids, or phosphoinositides have been found (Farooqui et al., 1997; Martín et al., 2010; Fabelo et al., 2012, 2014; Cheng H. et al., 2013; Naudi et al., 2015; Marin et al., 2017; Emre et al., 2021). In particular, the highly polyunsaturated docosahexaenoic acid shows a protective effect against AD in model systems, not only because of its ability to affect membrane fluidity but also by serving as a substrate for the formation of pro-survival and anti-inflammatory products (Hashimoto et al., 2011; Yang et al., 2011; Janickova et al., 2015; Belkouch et al., 2016; Zhang et al., 2018; Huang et al., 2019). However, it is not clear whether changes in the fatty acid and lipid composition of neuronal membranes are the cause or corollary of AD-associated brain deterioration.

### Cholesterol and GM1 cooperate in mediating Aβ neurotoxicity

Although cholesterol plays an important role, gangliosides, the sialic acid-containing sphingolipids, may serve as the fundamental Aβ-membrane interaction platform on which the monomeric form of Aβ self-aggregates into Aβ oligomers (Kim et al., 2006; Mao et al., 2010; Matsubara et al., 2017; Ahyayauch et al., 2020; Fantini et al., 2020; Rudajev and Novotny, 2020). In AD brains, GM1 and cholesterol have been shown to accumulate in nerve terminals where Aβ is concentrated (Gylys et al., 2007). Yanagisawa et al. observed the binding of Aβ to GM1 clusters that were dependent on cholesterol-induced GM1 aggregation. The authors concluded that microdomains rich in GM1 and cholesterol may be the site where Aβ accumulates and exerts its neurotoxic effects (Yanagisawa, 2005; Matsubara et al., 2017). Toxic, β-sheet-rich amyloid fibrils formed only when Aβ interacted with GM1- and cholesterol-rich membranes, but not in solution, or when the membrane lacked cholesterol and cholesterol-dependent lipid rafts (Okada et al., 2008; Matsuzaki, 2014; Ahyayauch et al., 2020).

Both Aβ40 and Aβ42 bound to cholesterol-dependent GM1 clusters and underwent a conformational transition from an α-helix-rich structure to a β-sheet-rich amyloidogenic conformation, but it was 10-fold more significant for Aβ42 than for Aβ40 (Matsuzaki, 2011; Matsubara et al., 2018). During early AD pathology, blockage of the endocytic pathway leads to cholesterol-mediated GM1 accumulation in early endosomes, where it serves as a platform for amyloid binding (Yuyama and Yanagisawa, 2009).

If aging is associated with increased cholesterol levels in synaptosomal membranes (Igbavboa et al., 1996) and more rigid lipid rafts (Martín et al., 2010; Fabelo et al., 2014; Diaz et al., 2015, 2018), age-related increased GM1 clustering could be a risk factor for the development of AD (Matsuzaki, 2014). Intense interactions between Aβ42 and cholesterol-containing GM1 clusters were key to accelerating Aβ fibrilization in exosome-like vesicles (Dai et al., 2020). Depletion of cholesterol and gangliosides significantly reduced Aβ-induced toxicity in both PC12 and SH-SY5Y cell lines (Wang et al., 2001). Isothermal titration calorimetry and Langmuir balance showed pronounced binding of Aβ42 monomers and oligomers and their incorporation into artificial model membranes composed of phospholipids, SM, cholesterol, and various types of gangliosides, including GM1. This interaction was followed by GM1- and cholesterol-dependent membrane destruction (Nicastro et al., 2016; Ahyayauch et al., 2021). Raman spectroscopy of GM1/SM/cholesterol-supported planar lipid bilayers tracked the binding of Aβ40 to the GM1-containing membrane, leading to membrane disruption. The N-terminus of Aβ40 remained in the vicinity of the polar lipid head groups, whereas the C-terminal fragment was inserted into the bilayer. During a 24-h incubation, the Aβ40 aggregated and changed its conformation from a random coil through an α-helix to a β-sheet structure (Hu et al., 2015). Increasing GM1 and cholesterol content in lipid bilayers facilitated Aβ binding to membranes. After binding to cholesterol-dependent GM1 clusters, Aβ underwent a conformational change from helix-rich to β-sheet-rich structures (Kakio et al., 2001). Hence, elevated cholesterol levels during aging could be a risk factor for Aβ toxicity in AD because amyloid concentration on lipid and protein platforms leads to peptide aggregation into the most toxic β-sheet-rich forms (Matsuzaki and Horikiri, 1999; Kakio et al., 2001, 2002; Rudajev and Novotny, 2020).

**Figure 3:**
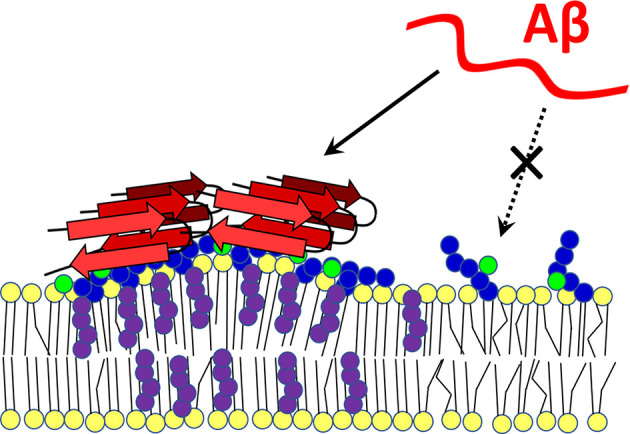
Cholesterol-dependent GM1 clustering and Aβ-membrane binding. Ganglioside GM1 was found to form membrane clusters in a cholesterol-dependent manner. Aggregated GM1 exists in a different spatial configuration that favors Aβ binding. With its -OH group, cholesterol forms a hydrogen bond with a sugar moiety of the polar group of the ganglioside, which positions it parallel to the membrane. When not bound to cholesterol, the polar head of the lipid is less able to interact with the amyloid peptide (dashed arrow). Red—amyloid β, yellow—membrane lipids, purple—cholesterol, blue—sugars in GM1 polar head, green—sialic acid. The scheme was prepared according to Yahi et al. (2010) and Matsuzaki (2014).

Amyloid peptides contain binding sites for both cholesterol and GM1. The Aβ-membrane interaction begins with the formation of electrostatic interactions of the basic amyloid residues with the negative charge of sialic acid on gangliosides, whereupon the peptide inserts into the hydrophobic zone of the bilayer with the help of cholesterol molecules. Then, Ca^2+^-permeable pore formation may occur (Lin et al., 2008; Di Scala et al., 2016; Venko et al., 2021). In another study, Aβ monomers bound to raft-like dipalmitoyl-PC:Chol:GM1 membranes and were incorporated into the bilayer only when both GM1 and cholesterol were present. In cholesterol-free DPPC:GM1 membranes, nascent Aβ binds to surface portions of gangliosides but does not penetrate the membrane (Rondelli et al., 2020). The concentration of GM1 and cholesterol in lipid rafts significantly increase the local density of amyloid-binding receptors, which can affect the secondary, tertiary, and quaternary conformation of amyloid (Fantini and Yahi, 2010). MD-based modeling has demonstrated that cholesterol can change the orientation of the polar heads of glycosphingolipids (GSL), which has a major impact on the Aβ-lipid interaction (Figure 3). The OH group of cholesterol forms an H-bond with galactose, which stabilizes the polar group of a GSL in a position parallel to the bilayer. If the polar group of a GSL is not stabilized in this parallel orientation, its ability to interact with Aβ is significantly reduced (Yahi et al., 2010).

### The role of membrane lipid composition in Aβ binding and toxicity

Fluorescence correlation spectroscopy and MD studies showed a strong dependence of Aβ40 aggregation on SM and GM1 concentration but not on cholesterol content (Amaro et al., 2016). SM has been proposed as a factor inducing the formation of surface-localized, β-sheet-rich toxic amyloid aggregates by reducing bilayer fluidity, as Aβ does not readily enter into the rigid bilayer formed by SM (Owen et al., 2018). Amaro et al. (2016) suggested that the Ld phase represents the physiological state in which Lo phase is absent. However, this is in stark contrast to a variety of other studies as well as the fact that cellular membranes are a mixture of lipids and proteins that stabilize Lo membrane domains (Anderson and Jacobson, 2002; Molander-Melin et al., 2005; Lingwood and Simons, 2010; Martín et al., 2010; Rushworth and Hooper, 2010; Hicks et al., 2012; Fabelo et al., 2014; Diaz et al., 2015; Arbor et al., 2016). Moreover, there is a difference in the interaction of Aβ40 and Aβ42 with membranes, as Aβ42, but not Aβ40, bound to Ld-phase liposomes formed from 1-palmitoyl-2-oleoylphosphatidylserine, POPC, and 15% cholesterol. The addition of SM led to the formation of Lo phase and increased Aβ40- but decreased Aβ42-membrane association (Carrotta et al., 2021). Fluorescence colocalization experiments on lipid vesicles revealed that Aβ42 was preferentially bound to and incorporated into the Ld phase. However, when GM1 was present, ganglioside reduced Aβ42 penetration by sequestering it to the polar head surface (Staneva et al., 2018).

In the AFM study, no interaction between Aβ42 and cholesterol itself was detected, but the simultaneous presence of ganglioside GM1 and cholesterol promoted oligomeric Aβ42 membrane binding and rapid destruction of the bilayer by a detergent effect. Thus, the formation of cholesterol-dependent GM1 clusters and the acceleration of GM1-Aβ42 cluster assembly may represent a mechanism of cholesterol-induced amyloid toxicity (Williams and Serpell, 2011; Ewald et al., 2019).

In addition, the presence of GM1 or other factors may drastically affect the resulting arrangement. According to many studies (Arispe and Doh, 2002; Curtain et al., 2003; Qiu et al., 2011; Evangelisti et al., 2014; Fernandez-Perez et al., 2018), cholesterol can play a protective role, but in combination with GM1, it becomes a toxicity-promoting agent (Matsuzaki and Horikiri, 1999; Kakio et al., 2001, 2002; Yanagisawa, 2005; Nicastro et al., 2016; Matsubara et al., 2018). Similarly, GM1 is known to be a neuroprotective molecule (Svennerholm, 1994; Svennerholm et al., 2002; Sokolova et al., 2007) but cholesterol-induced GM1 aggregation is responsible for its negative effect in AD-related neurodegeneration (Matsuzaki and Horikiri, 1999; Kakio et al., 2001, 2002; Yanagisawa, 2005; Bucciantini et al., 2014; Nicastro et al., 2016; Matsubara et al., 2018). Moreover, since amyloid peptides can bind to membranes with an artificial composition including simple phospholipids and without SM, GM1, or cholesterol (Dante et al., 2006; Brown and Bevan, 2016, 2017; Pannuzzo, 2016; Jamasbi et al., 2017; Karimi et al., 2019), it is very complicated to establish a realistic description of Aβ effects on physiological neuronal membranes.

## Conclusion

Cholesterol is one of the most ubiquitous lipid molecules in neurons, making it a potent modulator of cellular processes. This unique molecule accounts for up to 30% of the lipid molecules in the plasma membrane. It is therefore not surprising that most of the proteins and processes that take place in the PM are more or less dependent on cholesterol. This effect is underlined by the fact that cholesterol is responsible for the formation of lipid rafts, where many vital functions of the cell are localized and regulated. On the other hand, in Alzheimer’s disease, Aβ peptides are produced primarily as soluble molecules, and their association with cholesterol may represent a highly pathological event.

In the context of AD, cholesterol plays many roles. Cholesterol may be one of the protective mechanisms that strongly influence Aβ-membrane interaction and Aβ-induced bilayer disruption. Similarly, cholesterol may enhance the toxic effect of amyloid, as shown in many studies. The deleterious effect of cholesterol is associated with the localization of amyloidogenic APP processing in cholesterol-rich lipid rafts. Therefore, the precise cholesterol distribution within cell membranes, including various organelles or lipid rafts, may have implications for AD-related amyloid pathology. The distribution of cholesterol depends not only on its synthesis but also on intercellular and intracellular transport mediated by ApoE, ABC-transporters, receptors for lipoproteins, and cholesterol-modifying enzymes such as acyl-coenzyme A: cholesterol acyltransferases (ACATs) or cholesterol oxidases.

Since some authors have not confirmed the necessity of cholesterol for Aβ-membrane interaction (Kim et al., 2006; Amaro et al., 2016; Karimi et al., 2019), this suggests a strong dependence of the amyloid effect on the specific membrane composition. Because studies on the consequences of Aβ-cholesterol interaction provide conflicting results, one of the possible explanations could be that different amyloid forms, e.g., monomers, small oligomers, or larger aggregates of globular or fibrillar shape may behave differently. In several studies, cholesterol has been found to promote or hinder Aβ-membrane incorporation, which is often associated with the formation of calcium-permeable pores (Fabiani and Antollini, 2019). On the other hand, cholesterol-assisted incorporation of amyloid monomer into the membrane may reduce amyloid toxicity mediated by Aβ aggregation at the membrane surface (Qiu et al., 2009). Furthermore, as mentioned above, there are differences between Aβ40 and Aβ42 in their ability to bind to the membrane and cause cytotoxicity. Albeit also toxic, the truncated version Aβ(25–35) is often used, but it is very likely that this amyloid form exhibits different behavior than native amyloid peptides.

The specific lipid environment has a dramatic effect on the cholesterol-dependent binding and conformation of amyloid. This fact is extremely important when considering the results of MD studies or model membranes that use nonphysiological and artificial lipid ratios and often omit many lipids completely, including the highly variable sphingolipids. Therefore, MD simulations, studies on model membranes, and various *in vitro* and *in vivo* experiments may yield conflicting results (Avdulov et al., 1997; Qiu et al., 2011; Yu and Zheng, 2012; Dies et al., 2014; Fantini et al., 2014; Liu R. Q. et al., 2015; Amaro et al., 2016; Nicastro et al., 2016; Pannuzzo, 2016; Brown and Bevan, 2017; Henry et al., 2018; Staneva et al., 2018; Azouz et al., 2019; Ahyayauch et al., 2021). In addition to the specific lipid composition, membrane proteins also serve as Aβ receptors and their function is usually cholesterol-dependent, directly or indirectly through their association with membrane lipid rafts (Chen et al., 2017; Wiatrak et al., 2021). Nevertheless, the proteins are largely excluded from studies on amyloid-lipid membrane interactions, even though they essential contribute significantly to lipid raft formation by sequestering cholesterol with their cholesterol-binding domains (Lingwood and Simons, 2010; Grouleff et al., 2015; Fantini et al., 2016).

Although the role of cholesterol in AD pathology can hardly be disputed, the exact conclusion has yet to be drawn. The above-mentioned results of multiple studies indicate a strong dependence of the effect mediated by the cholesterol-Aβ interaction on all the players influencing cellular cholesterol levels and distribution, the lipid composition in the Aβ neighborhood, and the overall cellular context as well as the spatial arrangement of the amyloid itself. Even small changes in any of these parameters, including increases or decreases in the concentration of amyloid and cholesterol, gangliosides, sphingomyelin, or PUFA that occur during aging, can lead to shifts in the balance of Aβ production, degradation, export, oligomerization, and membrane binding. It must be reiterated that Aβ is an inherently unstable molecule whose conformation is highly dependent on the environment. It is therefore not surprising that any change in physiological conditions can lead to increased amyloid aggregation in the most toxic species and the development of AD.

In future studies, detailed and sophisticated analyses of complexes containing Aβ and specific membrane environments are in great demand. On the other hand, it is very complicated to perform such studies on human brains because they are not accessible for molecular analyses until after the death of the patient, when brain structure and function are altered to an extent that does not correspond to the earlier stages of the disease. Because of the somewhat different lipid composition in their brains, animal models can only provide us with partial information. Therefore, neural stem cells and brain organoids can help us uncover the mechanisms responsible for Aβ aggregating into the toxic forms upon contact with cell membranes and give us answers to the question of why the disease begins to develop in certain parts of the brain under certain circumstances.

## Author Contributions

VR performed the initial literature search and drafted the original manuscript. JN checked the cited data and revised the manuscript. Both authors contributed to the article and approved the submitted version.

## Funding

This work was supported by the institutional project SVV-260871/2020 (Přírodovědecká Fakulta, Univerzita Karlova).

## Conflict of Interest

The authors declare that the research was conducted in the absence of any commercial or financial relationships that could be construed as a potential conflict of interest.

## Publisher’s Note

All claims expressed in this article are solely those of the authors and do not necessarily represent those of their affiliated organizations, or those of the publisher, the editors and the reviewers. Any product that may be evaluated in this article, or claim that may be made by its manufacturer, is not guaranteed or endorsed by the publisher.
